# Lanthanides-Substituted Hydroxyapatite for Biomedical Applications

**DOI:** 10.3390/ijms24043446

**Published:** 2023-02-08

**Authors:** María del Carmen De Lama-Odría, Luis J. del Valle, Jordi Puiggalí

**Affiliations:** 1 Departament d’Enginyeria Química, Universitat Politècnica de Catalunya, EEBE, Av. Eduard Maristany 10–14, 08019 Barcelona, Spain; 2 Barcelona Research Center in Multiscale Science and Engineering, Universitat Politècnica de Catalunya, Campus Diagonal-Besòs, Av. Eduard Maristany 10–14, 08019 Barcelona, Spain; 3 Institute for Bioengineering of Catalonia (IBEC), The Barcelona Institute of Science and Technology (BIST), Carrer Baldiri i Reixac 11–15, 08028 Barcelona, Spain

**Keywords:** hydroxyapatite, cationic ions, doped HAp, lanthanidessubstitutions, biolabeling, cancer treatment, theragnostics, cell imaging, biomedicine, bone regeneration, implants, biosensors

## Abstract

Lately, there has been an increasing demand for materials that could improve tissue regenerative therapies and provide antimicrobial effects. Similarly, there is a growing need to develop or modify biomaterials for the diagnosis and treatment of different pathologies. In this scenario, hydroxyapatite (HAp) appears as a bioceramic with extended functionalities. Nevertheless, there are certain disadvantages related to the mechanical properties and lack of antimicrobial capacity. To circumvent them, the doping of HAp with a variety of cationic ions is emerging as a good alterative due to the different biological roles of each ion. Among many elements, lanthanides are understudied despite their great potential in the biomedical field. For this reason, the present review focuses on the biological benefits of lanthanides and how their incorporation into HAp can alter its morphology and physical properties. A comprehensive section of the applications of lanthanides-substituted HAp nanoparticles (HAp NPs) is presented to unveil the potential biomedical uses of these systems. Finally, the need to study the tolerable and non-toxic percentages of substitution with these elements is highlighted.

## 1. Introduction

In recent years, there has been an increasing demand for scaffolds, grafts, cements, fillers and implants in orthopedics and dentistry [1,2]. In order to satisfy this demand, different biomaterials have been considered for the elaboration of bioactive structures that could improve tissue regeneration, especially those that can guarantee short-term stability and long-term resorptive capacity [1,2]. Among these materials, synthetic stoichiometric hydroxyapatite (HAp) is preferred due to its biocompatibility and osteoconductive properties [1]. This is related to the fact that calcium phosphates represent 70% of the inorganic components of bone [2]. From a biological view, HAp can promote stem cell proliferation, migration and differentiation, alongside facilitating osteoblast adhesion and growth [1]. These biological effects facilitate the fixation of the implant to the host bone and improve mineral tissue growth at the bone–implant interface [3].

The limiting characteristics of HAp are its low mechanical properties and poor antibacterial activity. Regarding the first condition, HAp exhibits a reduced tensile strength, which restrings its use in bone grafts, fillers or coatings for a variety of implants [1]. Additionally, bone growth is strongly linked to angiogenesis, a process that HAp cannot directly stimulate and has been overcome by the loading of different growing factors into the mineral’s structure (e.g., VEGF, PDGF) [2]. Albeit the osteogenic and angiogenic effects of these signaling molecules, their activity can lead to ectopic or undesired bone formation [2].

In the case of scarce antimicrobial activity, pure HAp nanoparticles (NPs) cannot prevent the formation of biofilms on the surface of the material, which could lead to surgical failure. The most common cause of failure of orthopedic implants is bacterial infection from Gram-positive bacteria, such as *S. epidermis*, or from Gram-negative strains, such as *P. aeruginosa*. As these infections can be difficult to treat, some cases can only be solved by removal of the implant, which increases the cost of health attention and jeopardizes the life of the patient [1,4]. In this scenario, HAp doping elements are presented as an option to enhance the properties of minerals and improve clinical outcomes [1]. This approach helps imitate the conditions of biological apatite, which is normally found carbonated and substituted with trace amounts of ions [1]. On the other hand, some HAp dopants can play similar roles as growing factors [5]. The doped HAp NPs can be additionally repurposed as antimicrobial agents, drug carriers, bioimaging probes and theragnostics agents. Interestingly, recent research has mainly focused on the evaluation of substitutions with ions, such as Sr^2+^, Ag^+^ or Zn^2+^, forgetting the potential biological role of other cationic ions, such as lanthanides.

The present review aims to gather information on the effect of the incorporation of lanthanides on HAp’s biological and physical properties. In addition, this review provides information about their current use and prospects in the biomedical field. To attain this purpose, the article is divided into the following Sections: (Section 1) the characteristics of HAp; (Section 2) lanthanides-substitutions and their impact on the properties of HAp; (Section 3) the biological effects of HAp and the lanthanides; (Section 4) biomedical applications of lanthanides-substituted HAp; and (Section 5) discussion of the economic impact of lanthanides-substitutions on HAp synthesis. Section 4 is simultaneously divided into (Section 4.1) applications in orthopedical surgery and bone regeneration; (Section 4.2) applications in cancer therapy; (Section 4.3) development of NPs for the diagnosis, treatment or control of other pathologies; and (Section 4.4) lanthanides-substituted HAp for radiolabeling and bioimaging. To our knowledge, this is the first review to highlight the biological role of these elements and summarize their effect on the HAp structure.

## 2. Characteristics of HAp

HAp is a calcium phosphate with a stoichiometric formula of Ca_10_(PO_4)6_(OH)_2_ and a Ca/P ratio of 1.67 [6,7]. Two types of HAp crystal structures have been described: a monoclinic form, defined by the P2_1_/*b* space group (Z = 4), and a hexagonal form, defined by the P6_3_/*m* space group (Z = 2) [5,8]. In both structures, the PO_4_^−3^ anions arranged tetrahedrally along the *c* axis and the Ca^2+^ cations accommodate in the interstitial sites. The difference between them lies in the orientation of the OH^−^ groups. In the monoclinic structure, these groups are located in parallel planes to the *a*- and *c*- axes, but their orientation alternates between successive parallel planes [8]. The unit cell parameters of the monoclinic structure are *a* = 9.84214 Å, *b* = 2*a*, *c* = 6.8814 Å and γ = 120° [8]. An important characteristic of this form is that it can be easily destabilized by the presence of foreign ions [5].

The lattice parameters of the hexagonal structure are as follows: *a* = *b =* 9.418 Å, *c =* 6.884 Å, α = β = 90°, γ = 120° [8,9]. In this structure, calcium cations can be located at two different sites, Ca_1_ and Ca_2_. The Ca_1_ sites are those positioned in columns parallel to the OH^−^ channels while the Ca_2_ sites appear arranged in a triangular array. The mean Ca_1_-O bond length is 0.255 nm, whereas the mean Ca_2_-O bond length increases up to 0.245 nm. The phosphate groups are distributed in parallel with the crystallographic *c*-axis and with the channels in which the OH^−^ ions are located [6,7]. After dispersion of HAp NPs in aqueous media, there is dissolution of OH^−^ ions and exposure of Ca cations. The formed Ca sites are arranged on the *ac* or *bc* faces, which consequently become positively charged [10].

Respecting the electrochemical properties of HAp, it has been described as a purely proton-conducting material with conductivity values of 10^−9^–10^−7^ S/cm when temperatures are applied in a range from 500 to 800 °C. It must be considered that there is a difference in the conductivity behavior of individual HAp crystals and bulk sintered HAp ceramics. The latest possesses a relatively low proton conductivity attributed to grain boundary resistance to proton transport [11].

## 3. Impact of Lanthanide Incorporation on the Morphology and Properties of HAp

The calcium sites (Ca_1_ and Ca_2_) could be substituted by univalent, divalent, trivalent, tetravalent and pentavalent cations. The OH- group can be replaced by univalent anions that do not generate any charge imbalance, while the PO_4_^3−^ group can be substituted by bivalent anions that balance the charge of the calcium ions and hydroxide groups [12,13]. This is the case with biological apatite in which traces of elements such as Sr^2+^, Na^+^, K^+^, Zn^2+^, CO_3_^2−^, F^−^ or Cl^−^ can be found [5]. Due to substitutions, biological apatites have a lower degree of crystallinity, smaller crystal size, and higher solubility than stoichiometric HAp [14]. In addition, ion substitution leads to charge compensation either by uptaking a second substitution ion of an opposite charge or by creating vacancies in the crystal lattice [14,15].

The techniques reported for the synthesis of doped HAp include the most frequently used precipitation and hydrothermal methods [9,13,16,17,18,19,20,21,22,23,24,25,26,27], wet chemical precipitation method assisted by microwave reflux [4], sol-gel method/sol-gel microwave assisted/ultrasonic-assisted [8,28,29,30,31], electrostatic spray-pyrolysis [32], mechanochemical process [12], and magnetron co-sputtering [33,34].

In this Section, the changes in the HAp crystal parameters with increasing lanthanides concentrations will be discussed in detail. To facilitate the analysis of these changes, the data are summarized in Table 1 at the end of the Section. In some cases, the level of doping is explained as the value of variable *x* in the following formula:

Ca_10−x_ Dopant*_x_*(PO_4_)_6_(OH)_2_ (1)

In the particular case of terbium; this dopant was excluded from this review due to the scarce research that can prove their safe use in the body. With regard to ytterbium (Yb^3+^) and lutetium (Lu^3+^), albeit their inclusion in the biomedical applications section (Section 4), they were not considered for this and the following Sections (Section 3) due to the limited reports in the literature. The same criteria were applied to exclude Dy^3+^ and Er^3+^ from Section 3. Substitutions with promethium, holmium and thulium have not yet been reported.

### 3.1. Lanthanum (La^3+^)

Lanthanum (La) in the reaction solution affects the oriented growth of La-HAp crystals, which can be controlled by aging time and calcination temperatures. La-HAp crystals grow along the *a*-axis when the calcination temperature changes from 100 °C to 750 °C. Extending the aging time at 70 °C can favor the growth on the *c*-axis and applying hydrothermal treatment at 125 °C produces crystals with a low c/a value [23]. Furthermore, the incorporation of La into the crystal lattice can stabilize the apatite structure [35].

Pure and crystalline La-HAp NPs with a rod morphology can be obtained when *x* = 0.02 and the sol-gel method [28]. When increasing the *x* values to 0.06 and 0.1, higher crystallinity and crystallite size are described in both the sol-gel method and hydrothermal treatment [28,36]. Similar results were obtained by Ahymah et al., with doping values ranging from 10 to 50 mM [29].

**Figure 1:**
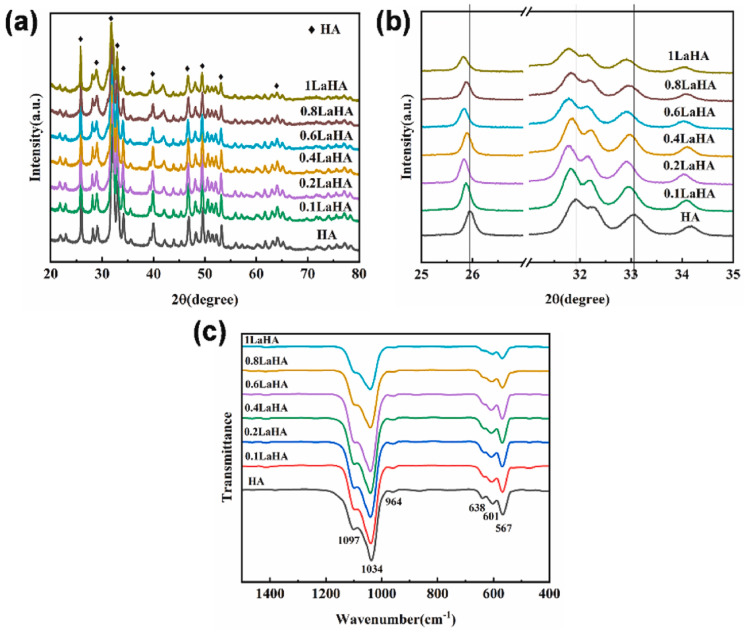
(**a**) XRD patterns of La-HAp with different La^3+^ content. In (**b**), a shift to the left of the XRD peaks can be observed with increasing La^3+^ concentrations, and in (**c**), the FTIR profile of the La-HAp powders with different La^3+^ content is depicted. Reproduced from [36] with permission from Elsevier. Copyright © 2023.

**Figure 2:**
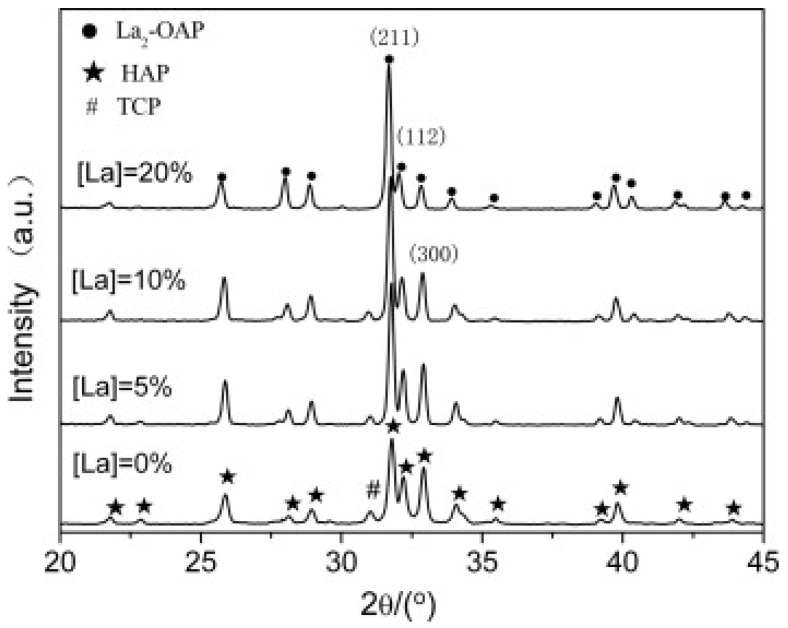
XRD patterns of the La-HAp with different La^3+^ content and prepared by a high-temperature solid-state reaction process. Peaks attributed to oxyapatite can be observed and a decrease of the diffraction planes of (112) and (300) planes is detectable with higher La^3+^ concentrations. Reproduced from [37] with permission from Elsevier. Copyright © 2023.

When analyzing XRD patterns, a decrease in the θ value and the peak shifts to the left were observed with increasing doping concentration as a consequence of the larger ionic radius of La^3+^ (0.106 nm) [36,37] (Figure 1 and Figure 2). The substitution also conditioned the zeta potential of the NPs, which gradually increased with higher doping percentages [36]. Ahymah et al. [29] and Lou et al. [38] described a gradually increased intensity of diffraction peaks with higher La^3+^ concentrations. These aspects can change based on the synthesis technique of HAp. For example, Guo et al. used a high temperature solid-state reaction process and observed diffraction peaks in La-HAp samples consistent with those from the La-incorporated OAP phase and the diffraction planes of (112) and (300) planes decreased with higher La^3+^ concentrations (Figure 2) [37].

Incorporation of this cation can similarly increase the surface area and hardness of the sample up to 31% and 14%, respectively. On the other hand, it can inhibit the dissolution rate of HAp in PBS [29].

### 3.2. Cerium (Ce^3+^/Ce^4+^)

Cerium (Ce) is the most abundant of the rare-earth metals in the Earth´s crust [39]. Depending on the chemical environment, Ce can present valence states at +3 and +4. The ionic radii of Ce^3+^ and Ce^4+^ are 0.114 nm and 0.097, respectively [30].

From the literature, most of the studies found of Ce-HAp NPs are based on Ce(III)-HAp and only a few analyze the crystallite changes induced by Ce(IV) doping. For Ce(III)-HAp made by the sol-gel route, XRD diffractograms show comparable peak positions to pure HAp with no significant shifts [40]. According to Kaygili et al., Ce(III)-doping values up to 0.5 at. % are associated with a slight increase in crystallinity [40]. Nevertheless, broader and less intense peaks can be detected with higher concentrations when techniques such as sol-gel and the microwave irradiation method are used. These changes express a decrease in crystallinity and smaller crystallites [25,26,30,31,41,42,43,44,45]. In the case of Ce(IV)-HAp, Paduraru et al. observed similar changes through the variation of the ion concentration from 0 to 10% [27]. Pathai et al. reported the presence of β-TCP diffraction peaks when an ultrasonic-assisted sol-gel technique was employed for the synthesis of Ce-HAp with a Ce molar fraction of 0.5 to 2 [30]. Kolesnikov et al. observed a slight shoulder at *2θ*~29° that corresponded to Ce-monazite when the NPs were synthesized by the precipitation method with a Ce/Ca ratio range from 0.05–5% [46]. Progressive increase of Ce^3+^ or Ce^4+^ leads to higher values of the *a* and *c* axes [27,47,48].

A decrease in the peak strength of the IR bands associated with P-O and O-H was detected with higher Ce concentrations [30,31,42,47]. In the case of changes in the O-H vibrational mode, it can be attributed to the creation of an electrical imbalance charge that will force the transformation of OH^−^ into O^2−^ ions [44]. When Paduraru et al. compared the IR spectra of the NPs, they found these bands to be decreased with a higher inclusion of the Ce^3+^ or Ce^4+^ into the HAp lattice until the molar fraction of 2.5% [27]. All of the aforementioned changes explained the increased solubility of the Ce-HAp NPs, as evidenced in the study by Padmanabhan et al. [31].

Changes in luminescence properties when doping HAp with Ce have been studied. Kolesnikov et al. determined that the optimal Ce/Ca atomic ratio was 0.3% when a precipitation approach for synthesis was considered. Increasing the doping ratio leads to a red shift of the emission maximum and reaches a maximum at a Ce/Ca ratio of 0.5%. Over this value, an internal quenching effect is described [46]. Paduraru et al. observed an increase in the absorption peaks and their broadening with increasing concentrations of the ions in the UV-Vis spectra of Ce(III)-Hap and Ce(IV)-HAp. In the photoluminescence emission spectra of the samples excited with 320 nm wavelengths, a decrease in the peak intensity was observed with higher doping percentages [27]. Based on these changes, Ce-doping can be proposed for cell probing and imaging. Similar results were described by Huang et al. [49].

### 3.3. Praseodymium (Pr^3+^)

Praseodymium (Pr) has an ionic radius of 0.100 nm and luminescent properties [50].

Agid et al. synthesized praseodymium-substituted HAp (Pr-HAp) NPs with various Pr^3+^ amounts of 2, 4, 6, 8 and 10 at. % by a wet chemical method, finding an increase in the crystallite size, the lattice parameter *a* and the unit cell volume. The crystallinity value gradually decreased with higher Pr^3+^ levels. No changes in the thermal stability of Pr-HAp were detected [51].

Ibrahimzade et al. co-doped HAp with Ce in a fixed amount of 0.35 at. % and Pr at increasing values of 0.35, 0.70, 1.05 and 1.40 at. %. In all cases, the formation of HAp, elaborated by a combustion method, was the major phase, and β-TCP was the minor one. It was concluded that the higher the Pr^3+^ concentration, the lower the HAp phase contribution. A continuous decrease in lattice parameter *a* and an increase in parameter *c* were also recorded. These changes presented together with a gradual decrease in the intensity of the IR peaks corresponding to the O-H vibrational modes [50].

### 3.4. Samarium (Sm^3+^)

Samarium (Sm), with an ionic radius of 0.095 nm, is an element exploited in medicine in the form ^153^Sm. This radioisotope is characterized by β and γ radiation emission. It has a half-life of 46.3h and an emission capacity of 0.29 MeV, which results in mean and maximal tissue penetration of 0.7 mm and 3.1 mm, respectively [52].

Sm induces changes in the XRD diffractograms of calcium phosphate when synthetized by a precipitation method. The diffraction peak intensity was reduced, and the peaks broadened with increasing concentrations. Interestingly, Sm-HAp NPs were also monodispersed in water [53].

Sm has an intense reddish-orange emission. Compared to europium(Eu^3+^) ions, Sm ions have a longer excitation wavelength with lower excited energy in the UV-Vis region and the main emission wavelength is shorter (601 nm vs. 621 nm of Eu^3+^). In Sm-HAp, the higher the Sm^3+^ concentration, a quenching effect can be observed due to non-radiative transitions and energy transfer of Sm^3+^ ions when synthetizing the NPs by a co-precipitation approach. The fluorescence lifetime (τ) decreases when Sm augments, which is the result of the decrease in distance and the enhanced interaction between Sm ions. The photoluminescence of Sm^3+^ in HAp can be affected by co-doping with strontium (Sr^+^) when Sr/Ca ratio varies in a molar ratio of 0 to 10 [54].

### 3.5. Europium (Eu^3+^)

In most of the reports, there have been no changes of XRD patterns, lattice parameters or the presence of other phases when incorporating Eu^3+^ by hydrothermal methods [55,56,57]. Victor et al. described a minor peak shift to larger angles when initial concentrations of Eu^3+^ (added at synthesis) corresponded to 0.5, 0.75 and 1 g. They also found a smaller crystal size and a decrease in the lattice parameter *a* when using a co-precipitation method [58]. As for the IR spectrum, samples can present a band at 1410 cm^−1^ attributed to CO_3_^2−^ (B-type substitution) and a band at 1510 cm^−1^ attributed to A-type substitution [59,60].

When prepared using the co-precipitation method, the Eu-HAp specimens showed a long nanowire microstructure. On the contrary, samples prepared by hydrothermal treatment exhibit a nanorod-like morphology that enhances luminescence due to the high packing density and reduction of light scattering [61]. Working at low temperatures, such as 120 °C, led to the ordering of Eu^3+^ ions at the Ca_1_ site [62]. Silva et al. observed that the thermal treatment of the doped NPs from 500 °C to 800 °C induces the diffusion of the rare element from Ca_1_ to Ca_2_ sites that seem to be more energetically stable to Eu^3+^ [63].

Based on its luminescence properties, Eu^3+^ complexes have been tested for cell bioimaging. That is the case of Eu(III)-β-diketonate and the chemically synthesized Eu(III) complex of dopamine and diethylenetriaminepentaacetic acid. The limitation of these systems is the degradation of color during cell observation [64]. The incorporation of the luminescent agent into HAp has been proposed to improve photo functions. When use as dopant, Eu^3+^ excited at 394 nm (or 460 nm) exhibits the following emissions: (a) when Eu^3+^ occupies the Ca_1_, a weak emission at 579 nm, 592 nm and 616 nm due to the ^5^D_0_ → ^7^F_0_, ^5^D_0_ → ^7^F_1_ and ^5^D_0_ → ^7^F_2_ transitions, respectively, can be detected; (b) strong emissions due to the ^5^D_0_ → ^7^F_0_ (574 nm), ^5^D_0_ → ^7^F_1_ (602 nm) and ^5^D_0_ → ^7^F_2_ (610–630 nm) transitions when occupying Ca_2_ [63]. Ciobanu et al. reported that the Eu^3+^ ions distribution on the Ca^2+^ sites did not modify the luminescence of the ion from the ^5^D_0_ → ^7^F_0_ transition was observed at 578 nm [65]. In agreement with these results, Liu et al. recorded an emission spectrum of Eu-HAp nanowires with a 5% of Eu^3+^ substitution and the dispersion solution presented emission signals ascribed to the ^5^D_0_ → ^7^F*_J_* (*J* = 1, 2, 3 and 4). The signal at 614 mn (^5^D_0_ → ^7^F_2_ electric-dipole transition) was stronger than the one located at 590 nm (^5^D_0_ → ^7^F_1_ magnetic-dipole transition) [56]. Nikolaev et al. determined that an Eu/Ca ratio of 2% was required for maximum luminescence [66]. Krishnapriya et al. reported similar results with eggshell-derived HAp NPs doped with Eu^3+^ [67]. A higher luminescent efficiency can be obtained by coordinating citric acid on Eu-HAp [68]. Albeit of the good results so far obtained, it must be considered that the doping of HAp with lanthanides for bioimaging can be limited by the concentration, as quenching effects can arise.

### 3.6. Neodymium (Nd^3+^)

Nd^3+^ has an ionic radius of 1.109 Å [69]. According to the XRD profiles of 1Nd-HAp, 5Nd-HAp, 10Nd-HAp and 20Nd-HAp elaborated by an ion-exchange method, only the latest presented a HAp phase and a Nd(OH)_3_ phase (18%). The presence of this phase was corroborated by the appearance of FTIR bands at 3609 cm^−1^ and 680 cm^−1^, which are attributed to the OH- stretching and libration mode in Nd(OH)_3_, correspondingly. Nd^3+^ improved the electrical conductivity, with the maximum value registered with 10Nd-HAp [70]. With different Nd^3+^ concentrations added by the precipitation method, a reduction in NP size was reported [71,72]. The crystallinity of the NPs can be reduced after 4 h of calcination at 700 °C of NPs synthesized by the sol-gel method and with increasing Nd^3+^ concentrations [69].

### 3.7. Gadolinium (Gd^3+^)

XRD profiles of 1Gd-HAp, 5Gd-HAp, 10Gd-HAp and 20Gd-HAp revealed the appearance of a second phase only with the highest Gd^3+^ concentration that corresponded to Gd(OH)_3_ (2.5%).These NPs were also capable of increasing the electrical conductivity 136 times higher than the pristine HAp [70].

Crystallinity can be hindered by the introduction of Gd^3+^ when working with molar ratios of Gd^3+^/Ca^2+^ from 0 to 0.10 and when using a liquid–solid–solution (LSS) hydrothermal method for synthesis. Amorphous NPs are formed with a ratio of 0.20 [73].

Annealing at 1100 °C leads to the formation of different phases when the Ca^2+^ substitution is in the range of 0.2 ≤ *x* ≤ 2. When *x* is up to 1.2, a Gd_2_O_3_ phase can be detected while GdPO_4_ and Gd_3_PO_7_ appear with higher values [74].

Gd^3+^ possesses a high magnetic moment as a consequence of the isotropic electronic ground state ^8^S_7/2_ and half-filled f-orbital with seven unpaired electrons [75]. Paterlini et al. developed Ca_9.80_Gd_0.20_(PO_4_)_6_(OH)_2_ NPs that showed strong luminescence [76]. It was also demonstrated that the addition of Gd to HAp leads to a shift in the thermoluminescence peak from 200 °C to 190 °C [77].

### 3.8. Dysprosium (Dy^3+^) and Erbium (Er^3+^)

Dy^3+^ can produce shifts to the right of the XRD peaks due to the small ionic radius (0.912 Å) that induces the formation of shorter Dy-O and Dy-PO_4_ bonds in NPs synthesized by the hydrothermal method [78].

Dy^3+^ ions have characteristic emissions at 480 nm and 575 nm due to ^4^F_9/2_ to ^6^H_15/2_ and ^4^F_9/2_ to ^6^H_13/2_ transitions. Dy-HAp NPs showed an intense blue emission favorable for bioimaging [79]. The thermoluminescence (TL) response of HAp NPs can be modified by Dy substitution with mol percentages of 0.5, 1 and 2, the second concentration being the one with the highest values [80]. Dy-HAp NPs even present a higher TL response than Ce-HAp, La-HAp and Gd-HAp [78].

Er^3+^ incorporation con generated changes in the unit cell volume, crystallite size and lattice parameters in a non-gradual trend when using a chemical process for synthesis [81]. Crystallinity is reduced with increasing concentrations of the element [82]. On the other hand, A- and B-type substitutions have been reported [82,83]. Under 400 nm light irradiation, Er-HAp exhibits emissions associated with purple, blue and green colors (^4^F_3/2_ → ^4^I_15/2_, ^4^F_7/2_ → ^4^I_15/2_, and ^4^S_3/2_ → ^4^I_15/2_ transitions, respectively) [83]. Table 1.

**Table 1:** Changes in the morphology and physical properties of HAp caused by lanthanides.

Lanthanides	Changes in HAp Morphology/Properties	Reference
Lanthanum	Low c/a value	[23]
	Higher crystallinity and crystal size (specially *x* = 0.06 and 0.1)	[28,29,38]
	Formation of second phases with increasing dopant concentrations and decrease in XRD peaks intensity of diffraction planes (112) and (300)	[37]
	Higher zeta potential values	[36]
	Increased specific surface area (31%) and hardness (14%), decreased dissolution in PBS	[29]
Cerium	Slight increase in crystallinity (0.5 at. %) and reduced crystallinity with higher values	[25,26,30,31,41,42,43,44,45]
	β-TCP diffraction peaks can appear with an ultrasonic-assisted sol-gel technique (molar fractions of 0.5 to 2)	[30]
	Decrease in FTIR bands of P-O and O-H	[30,31,42,47]
	Endows photoluminescence properties	[27,46]
Praseodymium	Appearance of β-TCP as a minor phase	[50]
	Increases crystallinity when Pr^3+^ values are 2,4,6,8 and 10 at. % and decreases with concentrations above the cited	[51]
Samarium	Monodisperse NPs, reduced crystallinity	[53]
	Endows photoluminescence properties	[54]
Europium	Favors A- and B-type substitution	[59,60]
	Nanowire structure when prepared by co-precipitation method and nanorod-like morphology when using hydrothermal treatment (enhancement of luminescence properties)	[61]
Neodymium	A Nd(OH)_3_ phase (18%) can be present in 20Nd-HAp NPs	[70]
	Particle size decreases with increasing concentrations of Nd^3^	[71]
	High doping concentrations can reduce crystallinity when calcinating at 700 °C for 4 h	[69]
Gadolinium	A Gd(OH)_3_ phase can be present in 20Gd-HAp NPs Increased electrical conductivity (136 times higher than HAp)	[70]
	Lower crystallinity with Gd^3+^/Ca^2+^ from 0 to 0.10 and amorphous NPs with a ratio of 0.20	[73]
	When *x* is up to 1.2, a Gd_2_O_3_ phase can be detected while GdPO_4_ and Gd_3_PO_7_ appear with higher values	[74]
	Endows photoluminescence properties	[75,76]
Dysprosium	Intense blue emission favorable for bioimaging	[79]
Erbium	Reduces crystallinity and favors A and B-type substitutions Er-HAp exhibits emissions associated with purple, blue and green colors	[82,83]

## 4. Biological Role of HAp and Its Substitution Ions

One of the most important applications of HAp and doped HAp is bone regeneration. Therefore, before explaining the biological effects of each element in the lanthanide series, the general concepts of bone biology are explained.

**Figure 3:**
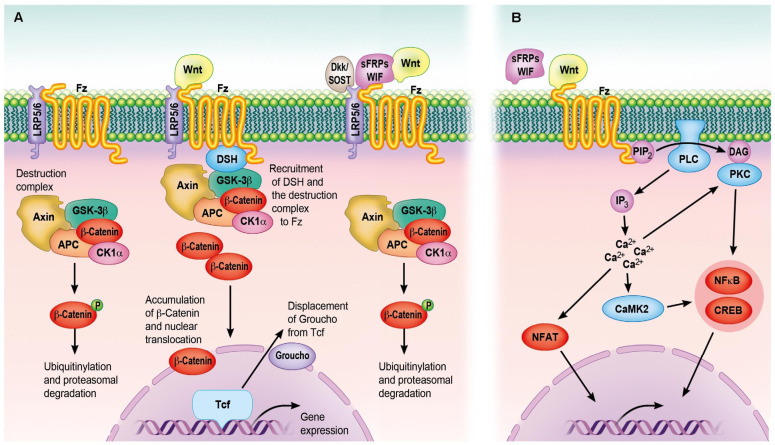
The Wnt signaling pathway. (**A**) Canonical Wnt signaling cascade. (**B**) Non-canonical Wnt signaling cascade. APC, adenomatous polyposis coli; CaMKII, calcium/calmodulin-dependent protein kinase type II; CK1α, caseine kinase 1-α; CREB, cyclic AMP-responsive element-binding protein; DAG, diacylglycerol; Dkk, Dickkopf; DSH, disheveled; GSK3β, glycogen synthase kinase-3 β; IP3, inositol 1,4,5-triphosphate; LRP, low-density lipoprotein receptor-related protein; NFAT, nuclear factor of activated T cells; NFκB, nuclear factor κB; PIP2, phosphatidylinositol 4,5-bisphosphate; PKC, protein kinase C; PLC, phospholipase C; sFRPs, secreted frizzled-related proteins; SOST, sclerostin; WIF, Wnt inhibitory factor. For more details on a molecular basis, articles [86,87] can be consulted. Reproduced with permission from [86].

The progression from an earlier stage to a more mature stage of osteoblast differentiation is characterized by the increased activity of alkaline phosphatase (ALP). ALP facilitates the beginning of mineralization by hydrolyzing organophosphates and releasing inorganic phosphates. Once the osteoblasts enter the mineralization stage, the levels of proteins associated with the maturation of the cells, such as ALP, decline and the expression of osteocalcin (OCN) increases together with the formation of HAp [84]. Both OCN and collagen type I (Col-I) are components of mature bone, and their detection indicates early mineralization of the extracellular matrix (ECM) [85]. To regulate osteoblast differentiation, Wnt/β-catenin pathway must be activated (Figure 3). It also regulates osteoblast attachment, differentiation, maturation, and apoptosis [85,86].

The implantation of biomaterials can trigger an inflammatory reaction that inhibits the osteogenic differentiation of endogenous stem cells. This immune response is commonly led by macrophages and their polarization into a pro-inflammatory phenotype (M1) that secretes pro-inflammatory cytokines, such as TNF-α or IL-1β. To circumvent this problem, regeneration scaffolds should regulate the host-to-scaffold immune response while exerting a pre-osteogenic environment. It is important that the biomaterial promotes the polarization of macrophages to the M2 phenotype, which can secrete anti-inflammatory cytokines and pro-osteogenic factors (e.g., BMP-2, VEGF) [88].

**Figure 4:**
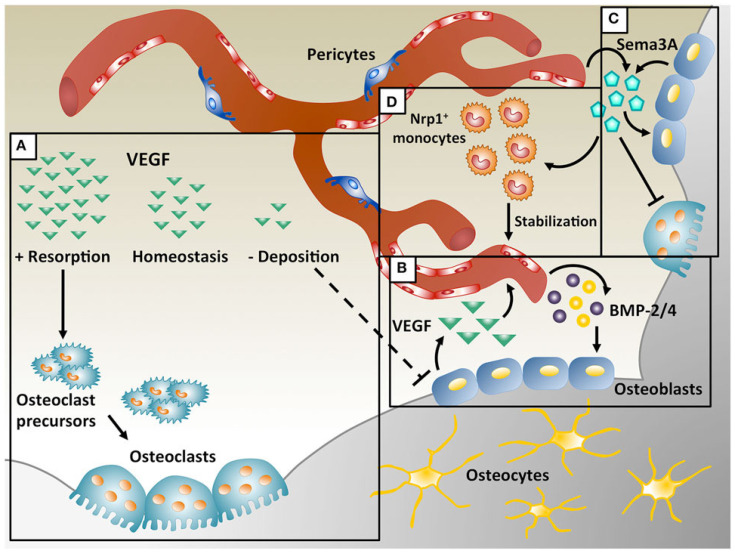
Association of angiogenesis and osteogenesis. (**A**) VEGF maintains bone homeostasis at physiological levels. Lower levels interrupt osteoblast differentiation and increase osteoclast recruitment. (**B**) Osteoblasts produce VEGF during bone repair, promoting migration and proliferation of endothelial cells. Endothelial cells later secrete BMP-2, supporting osteoblast differentiation. (**C**) VEGF regulates Sema3A expression, which suppresses osteoclast differentiation. (**D**) Sema3A is also responsible for the recruitment of neuropilin 1-expressing (Nrp1^+^) monocytes. Reproduced with permission from [89].

Bone remodeling is tightly linked to angiogenesis, which facilitates the transport of nutrients and gases to cells (Figure 4) [89]. In regenerative medicine, it is important that this migration of nutrients also reaches the cell located at the distal position of the implanted scaffold. With inadequate vascularization, a regenerative callus can be formed, resulting in an atrophic non-union of the bone [19]. To prevent this problem, different strategies have been followed: direct application of endothelial or progenitor cells into the scaffolds [90], development of scaffolds with a microfluidic network that acts as an analogue of natural vascularization [91], and genetic targeting of cells for enhanced synthesis of angiogenic growth factors [92]. Nevertheless, each of these presented disadvantages. In the first case, angiogenesis is a process that depends on an ample endothelial cell phenotype according to the tissue and these cells require the presence of other cells to control the vasculature formation [19,93]. In the second case, the technology involved can be highly sophisticated, costly or face limitations in the production of millimeter thick vascularized tissues that can directly anastomose with the host vascular system [91,94]. In the last case, the specific targeting of the host cells and the risk of introducing a vector gene into the patient translate into a difficult approach [92].

The events in bone regeneration can be controlled by the doping of HAp. For the regeneration of bone tissue, biomaterials should promote adhesion, spreading, cytoskeletal development and the formation of cell—matrix adhesion plaques [95].

When comparing the benefits of crystalline HAp NPs over amorphous calcium phosphate, the latest dissolves faster in vivo and exhibits poor adherence to the substrate. For these reasons, crystalline HAp is normally selected as an implant coating to favor durability and biocompatibility [11,96]. The NP size is another crucial factor in stimulating bone repairing. Studies on this matter have suggested that values below 100 nm can favor osteoblast adhesion, proliferation, ALP activity and calcium deposition due to the possibility of HAp NPs mineralizing the collagen fibers [96,97]. Exposure to the “a” and “c” planes of the hexagonal crystal structure has also been reported to influence osteoblast adhesion and subsequent bone formation. In detail, the “a” planes are rich in calcium ions, representing a surface to which the acidic group of proteins could bind. In contrast, the “c” planes promote the binding of the basic regions of the proteins [96]. Improvement in bone formation can be similarly achieved by the polarization of the HAp, specifically on the negatively charged surface of the crystal. When an electric field is applied at elevated temperatures, the crystals become polarized along the *c*-axis as a consequence of the field-induced movement of the protons. To obtain permanent electrical polarization, the electric field should be maintained as the crystals cool down [11].

Finally, fibroblast activity must be promoted as these cells synthetize ECM components and growth factors that assist in the integration of peri-implant soft tissue [95].

### 4.1. Lanthanum (La^3+^)

La can participate in stem cell differentiation, tissue regeneration and metabolism [85]. La^3+^ has anti-inflammatory, anti-hyperphosphatemic and osteoblast-enhancing effects. Lanthanum chloride (LaCl_3_) can inhibit the lipopolysaccharide (LPS)-mediated expression of pro-inflammatory cytokines and adhesion molecules in HUVECs by impairing the LPS-induced enrichment of NF-κB/p65 to the promoter regions of TNF-α, MMP-9, IL-1β, ICAM-1, and IL-6. A similar effect was observed over the enrichment of histone demethylase JMJD3 to the promoter regions of TNF-α, MMP-9, IL-1β, and IL-6. No cytotoxic effect on HUVECs was evidenced at concentrations ≤50 µM [98].

It has been detected that switching from calcium carbonate to lanthanum carbonate can delay vascular calcification in hemodialysis patients who require treatment for hyperphosphatemia [99].

La shows a positive phosphate-binding effect in bone metabolism [99]. LaCl_3_ can downregulate RANKL-induced NF-κB activity, NFATc1 activity and c-fos function, attenuating osteoclastogenic and bone resorption. Specifically, the downregulation of mRNA of cathepsin J, calcitonin receptor, and tartrate resistant acid phosphatase (TRAP) have been reported. In vivo, LaCl_3_ ameliorated the bone destruction induced by Ti particles in a murine calvarial osteolysis model [100]. LaPO_4_ can promote the osteogenesis activity of BMSCs via induction of Wnt/β-catenin pathway in vitro and in vivo. In the first case, increased ALP activity and extracellular matrix (ECM) mineralization are reported together with upregulated ALP, OCN and Col-I expression [85]. These NPs have low cytotoxicity and good intrinsic photoluminescence properties, so they can be used as orientation-sensing nanoprobes in bioimaging and microfluidics [85,101]. In addition, they have been reported as X-ray-mediated agents in tumor therapy [101,102].

The promotion of ALP activity, osteoblast adhesion and osteoblast proliferation can be similarly achieved with La-HAp NPs. Moreover, it has been demonstrated that these NP extracts do not have a cytotoxic effect on mouse fibroblast cells L929 [37].

### 4.2. Cerium (Ce^3+^/Ce^4+^)

Ce^3+^ has a similar size and bonding properties as Ca^2+^ so is capable of replacing the bication in the biomolecules [39]. It helps toinitiate the cell grip of integrins and it improves it [103].

It has been shown that CeO_2_ NPs can act as neuroprotectives and prevent cell oxidative stress when studies were performed on mouse hippocampal brain slices and the rodent cell line HT22, respectively. These studies have also highlighted the modulation of the brain-derived neurotropic factor (BDNF) pathway in a human Alzheimer’s disease model that can lead to neuronal survival. Bronchial epithelial cells have been shown to be more sensitive to treatment with these NPs. As negative effects of NPs, reduced liver weight, hepatocyte enlargement, reduced albumin levels and decreased potassium ratios in male Sprague-Dawley rats have been reported [39].

The antimicrobial activity of Ce(III)-HAp has been compared to that shown by HAp NPs [42]. The antibacterial activity of Ce(III)-HAp and Ce(IV)-HAp NPs against *S. aureus* and *E. coli* has been described [25,44,47]. The reported antibacterial effect of Ce is higher against Gram-negative bacterial strains [44]. In another study, both 5Ce-HAp NPs and their suspensions exhibited a biocidal effect against *E. coli* and *C. albicans* after 72 h of incubation [24]. The antimycotic activity of Ce-HAp NPs was also reported by Iconaru et al. [48]. Padmanabhan et al. also observed an antibacterial effect against *P. aeruginosa* [31]. Based on the study of Lin et al., it can be proposed that the minimum molar ratio of Ce/[Ce + Ca] to achieve an antimicrobial effect should be 0.12 [42]. The antimicrobial activity of Ce-HAp can be further improved by co-doping with silicone (Si) [104] and magnetite (Fe_3_O_4_) [105]. In the first case, Priyadarshini et al. found that Ce-HAp and Ce/Si-HAp could inhibit the growth of *S. aureus*, *B. subtilis*, *E. coli* and *P. aeruginosa* [104,106]. Similarly, adding Ce as a second dopant in Ce^3+^/Sr^2+^-HAp improves the antibacterial capacity of Sr^2+^-HAp when confronted with *E. coli* and *S. aureus* [43].

As explained in Section 2, Ce-HAp exhibits luminescence properties that are promising for the application of NPs in cell imaging and probing. Nevertheless, Huang et al. reported that the fluorescent of Ce-HAp (5 at. %) can suppress the proliferation of L929 cells at a dose higher than 0.1 mg/mL. In concentrations lower than the mentioned, no cytotoxic effect was evidenced after 12 h of incubation, indicating that this could be the correct dose when using NPs for cell imaging purposes [49].

In other cases of co-doping, albeit of not impacting the morphology, thermal stability, harness or crystallinity, the concentration of the second dopant can make the NPs unsuitable for biological applications. For example, a Yb^3+^ content over 0.56 at. % can reduce the viability of L929 cells to below 76% after only 24 h of incubation [107].

### 4.3. Praseodymium (Pr^3+^)

Pr can substitute Ca in the cell membranes and in the protein structure due to its similar ionic radius [50]. In addition to this knowledge, the biological function of Pr remains unclear, albeit its use in medicine for its radioactive isotopes _59_Pr^141^ or _59_Pr^140^ as a DNA binding probe for spectroscopic studies [108]. Of interest, Bhanjadeo et al. observed that 10 mM of PrCl_3_ are required to initiate a B-Z transition and maintain a stable conformation of the Z-bDNA conformation [108].

Yadav et al. reported the anti-tumor activity of Pr complexes on the human leukemia cell lines HL60 and Jurkat. The IC_50_ concentration was determined to be around 5 µM, and the results showed a significant increase in intracellular ROS coupled with an increase in the fraction of cells with sub-diploid DNA. Caspase 2 and 3 were activated, there was a translocation of Smac/DIABLO to the cytosol and the release of cytochrome C from the mitochondria was observed. Altogether, the authors concluded that the anti-tumor activity of the rare element was associated with ROS production [109].

In another report, Pr was used to improve the corrosion resistance and cytocompatibility of TiN coatings in vitro. Treatment of the endothelial cell line EAhy926 with the samples revealed increased proliferation on day 6. In addition, the endothelial cells showed good spreading on the TinN coatings. The presence of Pr also reduced the hemolysis rate from values over 5% to a percentage below 3% [110].

Ibrahimzade et al. reported a significant decrease in viability in L929 cells with increasing Pr concentrations. In detail, 0.35Ce-0.35Pr-HAp, 0.35Ce-0.70Pr-HAp, 0.35Ce-1.05Pr-HAp, and 0.35Ce-1.40Pr-HAp samples showed a78.1 ± 9.3, 73.5 ± 7.8, 59.3 ± 9.7 and 63.2 ± 9.4 percent viability [50].

### 4.4. Samarium (Sm^3+^)

Sm-HAp NPs (molar concentrations of 0.02 and 0.05) were tested against Gram-negative *P. aeruginosa* and *E. coli* and Gram-positive *E. faecalis* and *S. aureus.* To inhibit the growth of the first, concentrations of both samples higher than 0.125 mg/mL were required. For *E. coli*, concentrations of 0.05Sm-HAp NPs in the range of 0.031–1 mg/mL were needed, while an inhibitory effect on *S. aureus* was observed by 0.02Sm-HAp NPs for all tested concentrations, and a high concentration of 0.05Sm-HAp NPs in the range from 0.125 to 1 mg/mL showed weak antibacterial activity [53]. Iconaru et al. reported an inhibitory effect of 0.05Sm-HAp against *E. coli* and *C. albicans* [111]. The antifungal activity was also obtained with the 0.05Sm-HAp NPs in suspension and not only with the coatings [112]. In fact, increasing the molar concentration of Sm up to 0.5 preserved antifungal activity against *C. albicans* when the coating was prepared and as a NPs suspension [113].

The good viability of hFOB 1.19 osteoblast cells after 12 h of incubation with 0.02 and 0.055Sm-HAp NPs was described [53]. HeLa cells also exhibited good viability when incubated on 0.05Sm-HAp coatings or suspensions [112]. Nica et al. tested the biocompatibility of Sm-HAp NPs with Sm molar concentrations of 0.05 and 0.1 and found that HGF-1 gingival fibroblast cells had a viability similar to the control after 24 h. Interestingly, no antimicrobial effect was seen with sample concentrations of 5 mg/mL when confronted with Gram-negative *P. aeruginosa* and *E. coli* and the Gram-positive *E. faecalis* and *S. aureus.* Nevertheless, antibiofilm activity was demonstrated by concentrations of 1.25 mg/mL [114].

### 4.5. Europium (Eu^3+^)

The antimicrobial activity of Eu-HAp against *S. aureus*, *E. faecalis* and *P. aeruginosa* has been reported. In detail, a concentration range of 31–1000 µg/mL and 125–1000 µg/mL of Eu-HAp samples (Eu values of 0.1–2 at. %) were required to inhibit the growth of *E. faecalis* and *P. aeruginosa*, respectively. The same concentration reported for *E. faecalis* was needed for the antibacterial effect against *S. aureus*. Eu-HAp NPs’ antibacterial capacity against *E. coli* and *K. pneumoniae* can be improved by co-doping with silver NPs [115]. The antifungal effect against *C. albicans* was demonstrated with 2 and 5 at. % Eu values [59,116]. In the last case, the adherence development of fungal biofilm after 24 h of incubation was inhibited. In addition, both the suspension of NPs and the thin films caused a significant reduction in CFUs [116].

Incubation of HEK293 cells with 25 or 100 µg/mL of Eu-HAp samples did not inhibit growth or proliferation after 24 and 48 h of exposure. In addition, changes in Hsp60, Hsp70 and Hsp90 expression were not detected when compared to the control [60]. MG63 seeded onto Eu-HAp discs made with samples with Eu/(Eu + Ca) between 0.5% and 1.5% did not show changes in proliferation when compared to the control [117]. In agreement with these results, Ciobanu et al. observed good viability of the osteoblastic cell line with a higher Eu concentration [116]. Liu et al. described a good viability of BMSCs after 1, 3, 5 and 7 days of incubations with Eu-HAp NPs (concentrations from 3.125 µg/mL to 12.5 µg/mL). Interestingly, they also observed a proliferation tendency with concentrations ranging from 25 µg/mL to 200 µg/mL. The positive effect on BMSC growth was accompanied by increased expression of ALP after 14 and 21 days of culturing. Runx2 and OCN expression also became stronger on day 21 [56].

Cell viability analysis was extended to human brain cancer cells (U87 cell line), pheochromocytoma (PC12 cell line), and nervous tissue cancer cells (N2a cell line) treated with different concentrations of Eu-HAp NPs (Eu^3+^ atomic percentage of 15). In every case, good viability was observed [67]. Ortiz-Gómez et al. evidenced the cell viability of B16-F10 murine melanoma cells incubated with Eu-ACP at a concentration of 250 mg/L [118].

Eu-HAp NPs functionalized with arginine were tested as potential gene carriers in vitro due to the positively charged nature of the NPs after functionalization with amino acids. These systems are capable of binding to DNA without affecting the viability of A549 cells in a concentration or time manner. The NPs were internalized into the cytoplasm and perinuclear of the cells [119].

A concentration of 3 at. % of Eu^3+^ showed a capacity to increase metabolic activity, increase cellular division, and induce the differentiation of progenitor cells isolated from adipose tissue toward bone and cartilage-forming cells. The promotion of osteogenesis and chondrogenesis is related to the enhanced synthesis and secretion of ECM proteins specifically required for those tissues (Col-1 and OCN for bone tissue and aggrecan and Col-2 for cartilage) [120].

### 4.6. Neodymium (Nd^3+^)

Viability of the human fetal osteoblast cells HFOB 1.19 was demonstrated with Nd-HAp, 5Nd-HAp, 10Nd-HAp and 20Nd-HAp NPs [70].

Nd-Ce-Mg-Zn-HAp NPs (1.25% for each substitution ion) showed high resistance to *S. aureus*, *S. epidermis*, *S. mutans*, *E. coli* and *C. albicans*. The viability of WRL68 cells was demonstrated, while a cytotoxic effect on the osteosarcoma cells MG63 was reported [121]. All these effects were also seen with Nd/Zn-HAp NPs [122].

### 4.7. Gadolinium (Gd^3+^)

**Figure 5:**
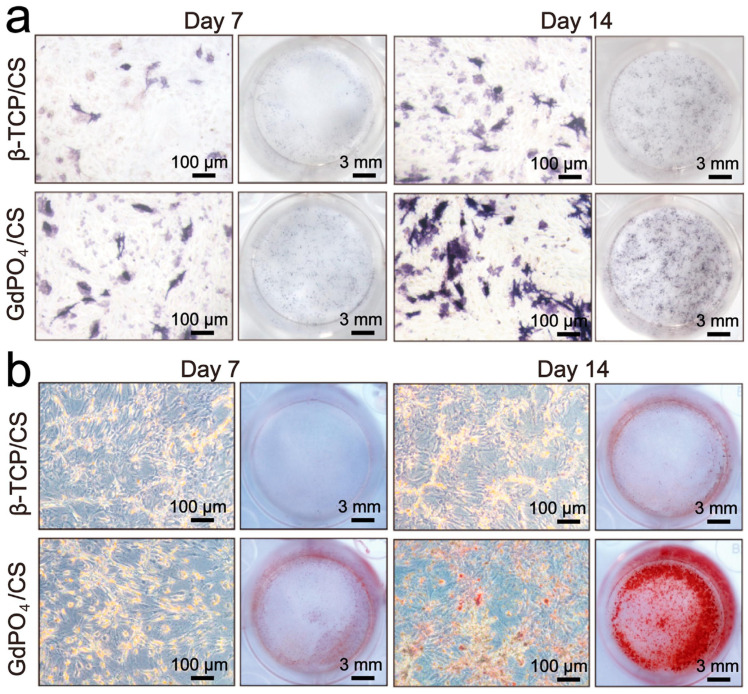
(**a**) ALP staining images and (**b**) alizarin red staining images of the rBMSCs co-cultured with the GdPO_4_/CTS and β-TCP/CTS scaffolds at days 7 and 14. Reproduced from [124], with permission from Elsevier. Copyright © 2023.

**Figure 6:**
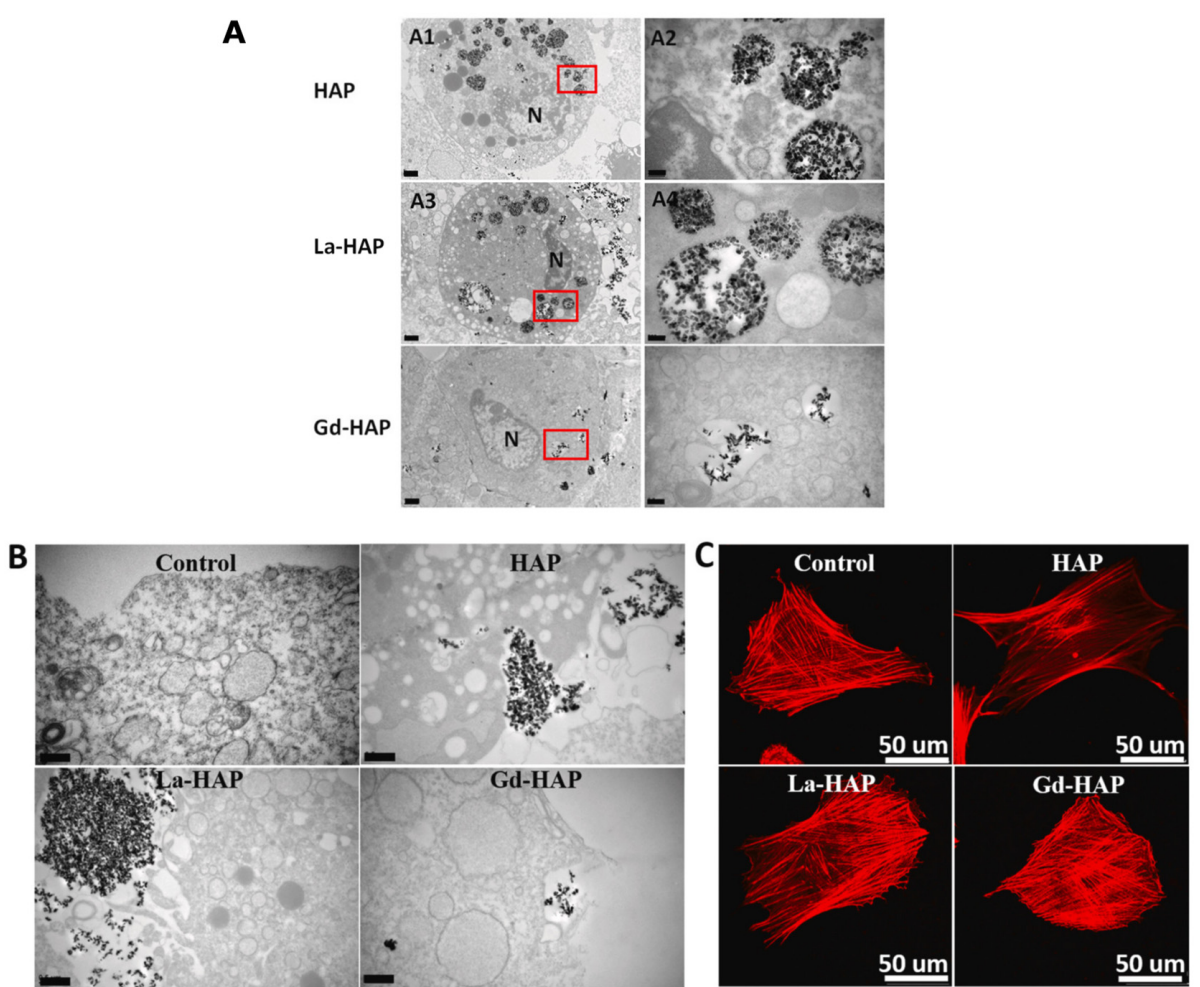
Uptake of HAp, La-HAP and Gd-HAP (the NPs were aforementioned as HAp, La-HAp and Gd-HAp, respectively) into BMSCs and F-actin morphology in the cells upon exposure to the NPs. (**A**) TEM micrographs of subcellular distribution of HAP (A1,A2), La-HAP (A3,A4) and Gd-HAP after 24 h. Overall cell morphology (left panel), scale bars: 1 μm. Higher magnification of cells in red-boxed areas (right panel, like A2 and A4); scale bars: 200 nm. (**B**) TEM micrographs of NP adhesion to the cell membrane following incubation with the NPs for 24 h; scale bars: 500 nm. (**C**) Confocal images of BSMCs showing the F-actin morphology in normal control cells and following incubation with the NPs for 24 h (Rhodamine-phalloidin stained actin filaments). Reproduced from [123] with permission from Elsevier. Copyright © 2023.

Gd participates in bone metabolism and is applied to the treatment of bone density disorders, such as osteoporosis [123]. The osteogenic differentiation triggered by Gd on BMSCs is controlled by the activation of the Smad/Runx2 signaling pathway. After this event, an upregulation of ALP, OCN, COL-I and RUNX-2 expression is described (Figure 5) [124]. Concentrations of up to 400 µg/mL of Gd-HAp can increase the proliferation of these cells. The internalization of these NPs when compared to pristine HAp or La-HAp showed a lower number of adhesion particles in the cell membrane and a fewer number of vesicles containing dispersed Gd-HAp NPs in the cytoplasm (Figure 6) [123].

Gd^3+^ can modulate lipid metabolism like La and Ce by inhibiting adipogenesis in 3T3-L1 preadipocytes. Furthermore, Gd^3+^ can promote cell growth and proliferation by accelerating the cell cycle entry into the S phase. This ion can also induce stronger sustained ERK activation at different stages of the cell’s differentiation [125].

Viability of the human fetal osteoblast cells HFOB 1.19 was demonstrated with Gd-HAp, 5Gd-HAp and 10Gd-HAp NPs, while cytotoxicity was observed with 20Gd-HAp NPs [70]. Biocompatibility has been similarly proven in MC3T3-E1 cells [126].

With regard to Gd-HAp hemolytic properties, the hemolysis percentage has been reported below 2% with concentrations up to 4 mg/mL and, based on the ASTM standard practice for assessment of hemolytic properties, they can be considered non-hemolytic. The integrity of the erythrocyte’s membrane after treatment has also been confirmed in SEM micrographs [127].

Gd^3+^ ions endow HAp NPs with antibacterial capacity against *E. coli*, *S. aureus* and *S. epidermidis* [126,128].

Nevertheless, the administration of Gd^3+^ can cause nephrogenic systemic fibrosis in patients with severely impaired kidney function, so special attention to tolerable concentrations of NPs should be paid [123].

## 5. Biomedical Applications of Lanthanides-Substituted HAp

### 5.1. Lanthanides-Substituted HAp in Orthopedical Surgery and Bone Regeneration

One of the main complications in the orthopedic field is implant-associated infection. The initial and crucial step is the aggregation of microorganism adhesins on the implant surface and subsequent biofilm formation. To address this problem, the encapsulation of antimicrobial agents in HAp has been tested. Nevertheless, the rapid emergence of antibiotic resistance and the fact that pharmaceutical agents, once released, are washed out by body fluids in a short time, mainly explains why thetreated area and the implant surfaces are left unprotected from long-term microorganism infections [129]. For this reason, HAp NPs are doped with different ions with antimicrobial properties. The preferred technique to coat the implant surface with the substituted HAp NPs is thermal spraying [130,131,132,133,134], but other techniques, such as magnetron sputtering [135], micro-arc oxidation [136], sol-gel coating [40,137], and alkali-thermal oxidation pretreatment followed by the use of a modified supersaturated calcification solution [44], can also be used. Two important disadvantages of magnetron sputtering are the low deposition rate and the amorphous character of the HAp NPs that lead to the post-deposition treatment of the materials by annealing, hydrothermal treatment or laser irradiation to improve the crystallinity [40]. Plasma spray deposition presents the disadvantage of forming HAp with lower crystallinity values and the formation of a variety of chemical phases due to high temperatures and rapid cooling. As already explained in Section 2 and 3, the presence of additional phosphorus compounds accelerates the dissolution of the mineral layer. To overcome this problem, new coating techniques, such as the room temperature process IonTite^TM^ are being studied [96].

Independent of the chosen coating technique, Ti-OH groups are required on the Ti surface to induce HAp nucleation. For this reason, an alkaline pretreatment (thermal immersion in NaOH solution) is sometimes carried out to form a bioactive sodium titanate (Na_2_Ti_5_O_11_) layer on the Ti implant surface [44]. This treatment could also be applied when coated with doped HAp.

Ti coating with La-HAp can offer some advantages in peri-implant osteogenesis. Lou et al. observed that the introduction of La into the apatite lattice made the implant coating more uniform, without visible micro-cracks and with a lower degradation rate. Good viability of MC3T3-E1 cells and promotion of osteoblast activity were described. Nevertheless, this cationic doping did not represent a significant increase in the bonding strength of the coatings, so other La^3+^/Ca^2+^ ratios could be included in future research [38].

**Figure 7:**
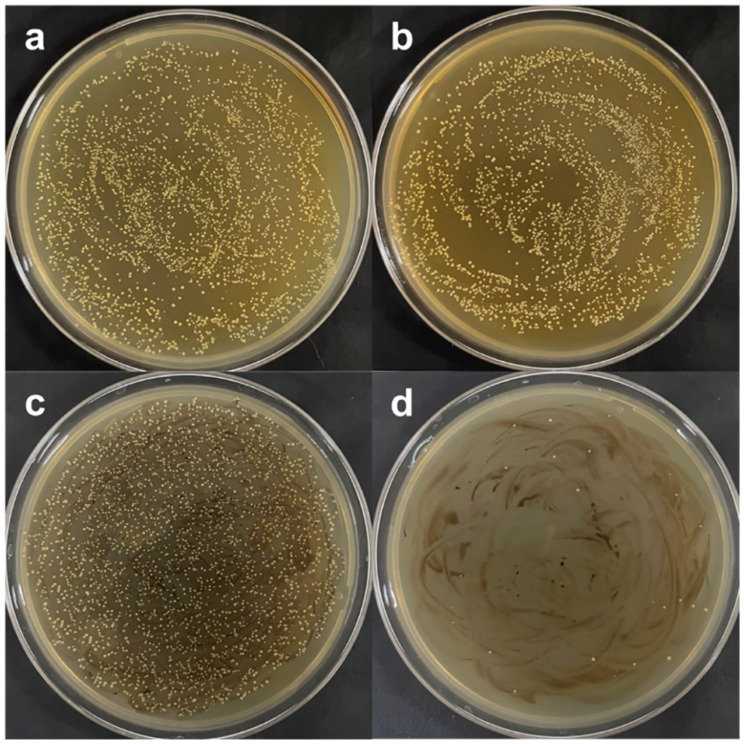
Colony plots of different samples after co-culturing with *E. coli* for 2 h. (**a**) HA13, (**b**) 1La-HA13, (**c**) 1La-HA13/PDA before irradiation, and (**d**) 1La-HA13/PDA after irradiation. NPs are referred in the review as HAp, 1La-HAp and 1La-HAp/PDA, respectively. Reproduced from [36].

Wang et al. proposed La-HAp NPs (with *x* values from 0.1 to 1) wrapped in poly-dopamine (PDA) as a biomaterial to apply photothermal therapy (PTT) and enhance antimicrobial activity. They concluded that PDA has a good photothermal effect and 1La-HAp/PDA has a significantly higher photothermal warming capacity. Specially, 1La-HAp/PDA produced at pH 13 revealed better photostability after 3 heating cycles with an 808 nm laser (2 W/cm^2^). The nanocomposite had a more significant release of Ca^2+^ ions and La^3+^ within the first 4 days. These release rates did not translate into increased cytotoxicity when tested on MC3T3-E1 cells. Finally, the application of laser radiation to the samples led to an antibacterial rate of 99.1% against *E. coli* (Figure 7). This is explained by the rapid heat up of the particles after irradiation, reaching a solution temperature of 50.9 °C that can denature the proteins from the bacterial membrane [36]. As better activity against *S. aureus* and *P. aeruginosa* has been reported for La^3+^/Sr^2+^ HAp NPs [138], further studies could be performed to incorporate the co-doped NPs into the same or other systems for PPT.

Ti implants can be coated with composites consisting of Ce-HAp and natural [44,139] or synthetic polymers [140]. In the first case, Ti implants were coated with Ce-HAp/collagen by a biomimetic method that required a modified supersaturated calcification solution after chemically modifying the surface of the implant by alkali and thermal oxidation. According to the authors, this procedure will facilitate the Ce^4+^ substitution of Ca^2+^ and the attraction of the positively charged collagen fibrils, which will act as nucleation reagents on the negative surface of the implant. Antibacterial tests revealed that 92.61% and 73.59% of *E. coli* and *S. aureus* were killed after 24 h. This effect could be associated with the decreased crystallinity of the doped HAp NPs, increasing the solubility and availability of Ce, which can interact with the cell membrane of bacteria and generate structural damage and cell death [44].

Dos Santos et al. developed a composite based on Ce-HAp and the biopolymers cashew gum and gellan gum for bone regeneration. The mechanical test of the materials showed a compressive strength of 19.19 MPa and a modulus of elasticity of 0.24 GPa. This matrix had the ability to absorb the ions from the PBS solution and the cell proliferation and growth were not affected by the composite [139].

**Figure 8:**
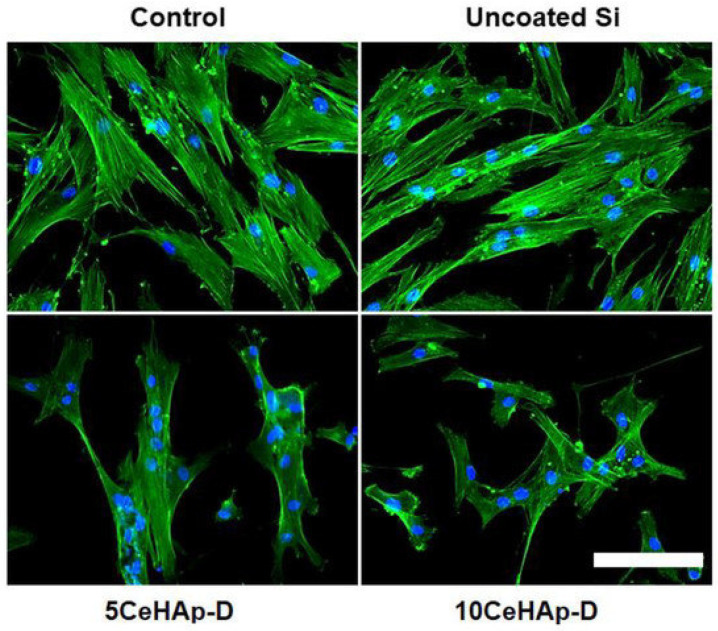
Actin cytoskeleton organization of gingival fibroblasts after 24 h of incubation with uncoated Si substrates and different dextran-coated cerium-doped hydroxyapatite coatings (5CeHAp-D and 10CeHAp-D). F-actin (green) was labeled with phalloidin-phalloidin-fluorescein isothiocyanate (FITC) and nuclei (blue) were counterstained with 4′,6-diamidino-2-phenylindole dihydrochloride (DAPI). Scale bar: 20 µm. Reproduced with permission from [141].

Composites of dextran and Ce-HAp (x = 0.05 and 0.1) were similarly tested as implant coatings, obtaining the attachment and proliferation of human gingival fibroblast cells (Figure 8). The cells retained their elongated morphology and effectuated multiple focal adhesions after 24 h of incubation [141].

Regarding synthetic polymers, there are reports of composites made with Ce-HAp and PLA [140] that offered the advantage of protecting HAp from decomposing to TCP when sintered.

**Figure 9:**
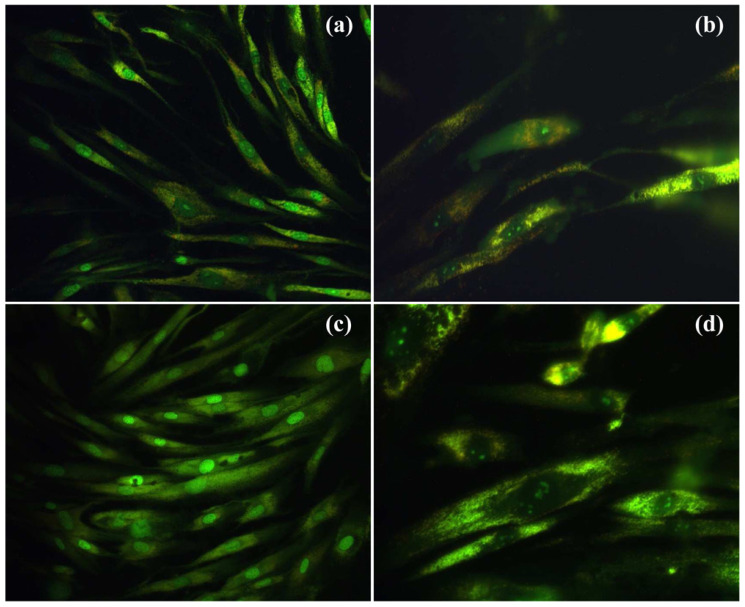
Adhesion of MC3T3-E1 cells on Sm/Gd-HAP coating after culture of (**a**,**b**) 5 days and (**c**,**d**) 7 days. (Green features correspond to vinculin in the focal adhesion complex.) Reprinted with permission from [143]. Copyright © 2023 American Chemical Society.

One more concern regarding the use of metallic implants is the associated release of constituent ions. This is the case of NiTi-shaped memory alloys that, albeit matching the mechanical behavior of bone, are characterized by the release of Ni, which not only weakens the osseointegration process but could also reach toxic concentrations. A recent proposal to control ion release is to coat the implant surface with doped HAp NPs [142]. For this purpose, implant corrosion should also be reduced. Priyadarshini et al. presented a triple-layer coating of Ce-HAp or Ce/Si-HAp over Ti-6AI-4V implants that exerted an anticorrosion effect while offering osteoconductive and antibacterial properties [106]. It was proposed that the released Ce in the SBF medium would form Ce(OH)_3_ that precipitated and protected the cathodic site, stopping the anodic reaction [106]. Sm/Gd-HAp NPs can also offer anticorrosive protection to borate-passivated AISI 316L SS stainless steel and a simultaneous antibacterial capacity against *S. aureus* and *E. coli.* A higher adhesion, viability and proliferation of MC3T3-E1 cells was additionally reported (Figure 9) [143].

Polypyrrole (PPy) is a conductive polymer that offers corrosion resistance. The incorporation of Sm-HAp and *Wrigthia tinctoria* (WT) fibers was carried out to create a biocomposite for Ti coating by electrophoretic deposition. The addition of WT fibers increased the adhesion strength of the biocomposite coating (20.04 ± 0.5 MPa), while the presence of PPy brought corrosion resistance to the coating (i_corr_ values of 0.37 ± 0.09 μA cm^−2^ when compared to 4.42 ± 0.4 of uncoated Ti and 1.58 ± 0.3 of only Sm-HAp coating). The antibacterial activity was enhanced against *E. coli* and *S. aureus* with maximum activity when 100 µL of a stock solution of 0.1 g/mL were used. The antifungal action of the coating was observed against *A. niger.* The viability of the composite was demonstrated on MG63 cells with volumes of 12.5, 25, 50, 100 and 200 µL of the mentioned stock solution. The authors obtained similar results when testing a composite made with poly(3,4-propylenedioxythiophene(PProDOT), Eu-HAp and *Calotropis gigantea* fibers [144].

Doping HAp can also help in the elaboration of cell-free tissue-engineered bone reconstruction scaffolds. The objective of these systems is to recruit endogenous stem cells while regulating the host-to-scaffold immune response, which can provide an osteogenic environment. For example, Wang et al. presented a magnetic La-HAp/chitosan (CS) scaffold capable of recruiting BMSCs, modulating macrophage polarization, and enhancing stem cell osteogenic differentiation in vitro and in vivo. The scaffold doped with magnetic M-type hexagonal ferrite (SrFe_12_O_19_) exerted an improved cell migration, attachment, survival and proliferation of rat bone marrow mesenchymal stem cells. Flow cytometry showed that after 24 h of culture on the magnetic La-HAp/CS scaffolds, the M1 macrophages exhibited a lower fluorescens percentage, corresponding to the marked CCR7. In contrast, the M2 macrophages showed a higher fluorescence of the differentiation marker CD206. These results were accompanied by increased ALP activity and regulation of the pro-osteogenic effect by Smad 1/5/9 pathway activation. In vivo, treatment of a rat calvarial defect showed that this scaffold favored the formation of more bone tissue than the controls, so it was concluded that the magnetic particles are responsible for mesenchymal stem cell recruitment and La^3+^ of the regulation of the macrophage’s phenotype [88].

Agarose/Gd-HAp composites were presented as artificial bone fillers. The 3D porous structure demonstrated an enhanced antibacterial effect, bioactivity and osteogenic properties when tested on MC3T3-E1 cells [126].

Combining Yb-HAp NPs with SrFe_12_O_19_ in a CS scaffold offers the nanocomposite the capacity to induce the in-growth of blood vessels and new bone into the 3D channels of the nanohybrid, accelerating bone healing [145].

Other scaffolds can be elaborated via 3D printing of bioinks, such as alginate, chitosan, fibrinogen or gelatin. Biomaterials can be modified to improve their mechanical properties and prevent fast degradation in physiological media. This is the case with gelatin modified by methacrylic anhydride, which obtains methacrylate groups for photo-crosslinking (gelatin methacryloyl or GelMA). Leu Alexa et al. further enriched this material with Ce-HAp NPs (5 at. %) to create a printable hydrogel with intricate architecture and higher structural integrity. The scaffold produced with a GelMA concentration of 30% demonstrated the highest viability and proliferation of murine preosteoblasts (MC3T3-E1) when compared to GelMA/HAp and other biomaterials produced with different Ce/Ca ratios. After 14 days of osteogenesis induction, there was a significant increase in the expression of OSX. After 28 days, higher OPN expression was registered [45].

Cryogelation is a technique that allows the formation of porous scaffolds with a controlled pore size. The technique consists of utilizing ice gems to create and control pores. Later, crosslinking was executed at temperatures below zero, obtaining an interconnected porogen structure after thawing. This method was employed to elaborate a degradable chitosan-gelatin/Ce-Zn co-doped HAp composite that could be used as a scaffold for bone-guided regeneration. The resultant material showed better protein adsorption, higher permeability and higher swelling effect than the composite made with pristine HAp. Higher bone marrow stromal cell proliferation after 3 days and higher ALP activity at days 1, 3 and 7 were reported [103].

### 5.2. Lanthanides-Substituted HAp for Cancer Therapy

As for cancer treatment, radioembolization is a method that consists of introducing particles of a solid carrier that entraps a radionuclide into a blood vessel that feeds the tumor. This therapy aims to kill the tumor by irradiation and by decreasing the tumor´s nutrient supplies. In a recent investigation, HAp prepared by the enzymatic hydrolysis of calcium glycerophosphate (enzymatic HAp) was used to carry ^90^Y, copper-67 (^67^Cu) and ruthnium-103 (^103^Ru). An irreversible sorption of ^90^Y and ^67^Cu that reached a maximum at 15 min and 1 h, respectively. Sorption values of ^103^Ru were significantly lower than ^90^Y. Nevertheless, studies of the biological effects of the system have not yet been performed [146].

Functionalization of lanthanides-substituted HAp with different polymers allows their application for theragnostics [58]. Doping with lanthanides can facilitate the creation of bimodal or tri-modal contrast imaging systems. In the first case, Dy-HAp was tested [147]. In the second case, doping with Eu^3+^ (3 at. %) results in a bright near-infrared fluorescence and co-doping with Gd^3+^ offers an improved paramagnetic longitudinal relaxivity and an 80% X-ray attenuation that are suitable for MRI and X-ray contrast imaging, respectively [148].

Victor et al. produced a composite with cyclodextrins and cucurbiturals (CB) to augment the drug-loading capacity of Eu-HAp, including hydrophobic drugs. The selected drug was the antitumoral agent 5-fluorouracil (5FU). They varied the Eu^3+^ concentrations by adding 0.5, 0.75 or 1 g of the Eu3+ reagent during synthesis (HEu0.5, HEu0.75, and HEu1, respectively). They observed that by increasing the doping concentration, they could obtain a higher loading efficiency of the drug. The drug loading decreases the luminescent intensity of the system, but the emission line at 620 nm appears in the emission spectrum and increases intensity with 5FU release, making cell monitoring possible. Finally, the samples without drugs depicted cell viability values greater than 80% when tested on HeLa [58].

**Figure 10:**
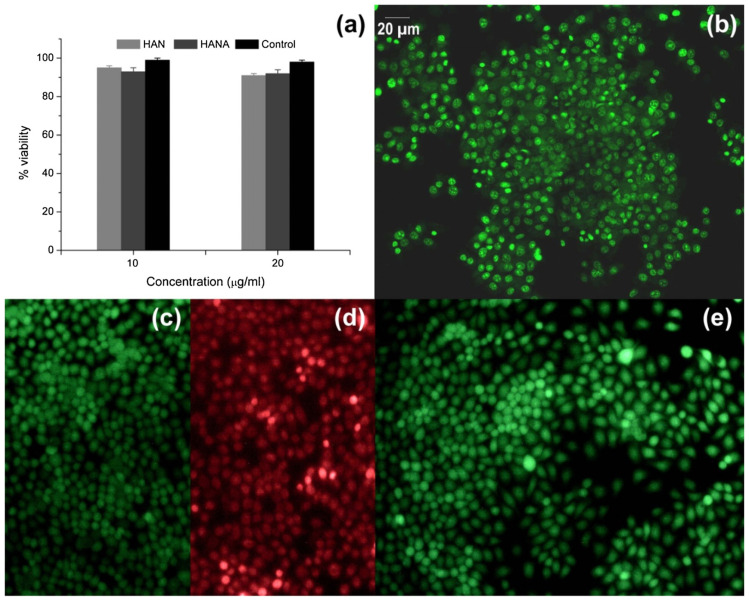
(**a**) MTT assay results. Live/dead image of cells treated with (**b**) Nd-HAp, (**c**) negative control, (**d**) positive control and (**e**) HAp NPs. Reproduced from [72] with permission from Elsevier. Copyright © 2023.

Nd-HAp NPs functionalized with cyclodextrin were tested as drug carriers of doxorubicin (DOX), obtaining better drug loading and release rates. The system with a positive zeta potential showed good uptake by C6 glioma cells and a near-infrared emission at 680 nm endowed by the Nd^3+^ ions that allowed their tracking [71]. Similar results were obtained with alginic acid/Nd-HAp composites loaded with 4 acetyl salicylic acid for the treatment of colorectal cancer. Alginic acid allowed augmented loading and pH-responsive release of the drug in more neutral and basic environments. Biocompatibility was demonstrated on L929 cells, and despite the quenching effect of the hydroxyl groups from the drug, the system sustained its theragnostics potential (Figure 10) [72].

**Figure 11:**
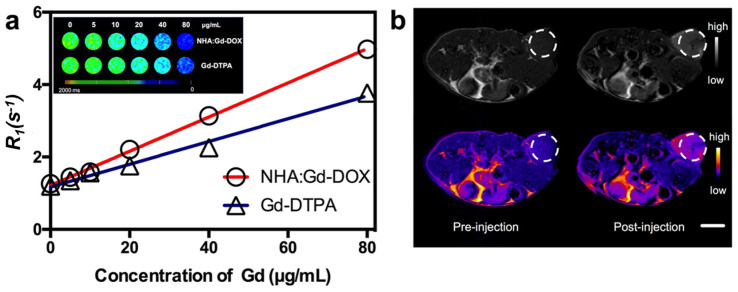
(**a**) r1 of DOX/Gd-HAp (indicated as NHA:Gd-DOX in the plot) and Gd-DTPA. Inset: T1 map MRI of different concentrations of NPs and Gd-DTPA. (**b**) MRI (above) and the color-mapped images (below) of mice post-injection and 15 min after injection of NPs at 10 mg/kg. Scale bar: 5 mm. Reprinted with permission from [73]. Copyright © 2023 American Chemical Society.

Gd-HAp NPs also showed potential for theragnostics, as they have a higher brightness on magnetic resonance contrast images than Fe-HAp or Co-HAp [127]. DOX was loaded into the NPs, obtaining a high loading capacity (118 µg/mg) and a pH-responsive release. The T1 relaxivity (r1) was higher than that obtained with the commercial T1 contrast GD-DPTA (Figure 11). When tested with MCF-7 cells, the magnetic resonance images became brighter with increasing concentrations of NPs. In vivo, there was a significant reduction in the tumor volume compared to free DOX [73]. Co-doping of the NPs with Ag^+^ can further improve the X-ray, CT and MRI results (25Ag/50Gd-HAp and 25Ag/75Gd-HAp NPs) [149].

Cipreste et al. made Gd-HAp NPs (1.3 and 2.8%) that were neutron activated (Gd to ^159^Gd and P to ^32^P, respectively) to induce beta and gamma activities. These can be used in multi-imaging diagnoses on single photon emission computed tomography (SPECT) and MRI systems. Finally, they functionalized the obtained NPs with folic acid for the active targeting of osteosarcoma cells [150].

Radioactive/magnetic ^153^Sm/Gd-HAp nanorods were produced and showed biocompatibility on HeLa at 48 h of monitoring with even concentrations of 800 µg/mL. SPECT and MRI of BALB/c mice treated with the NPs revealed a fast uptake by the mononuclear phagocyte system in the liver and spleen [75].

**Figure 12:**
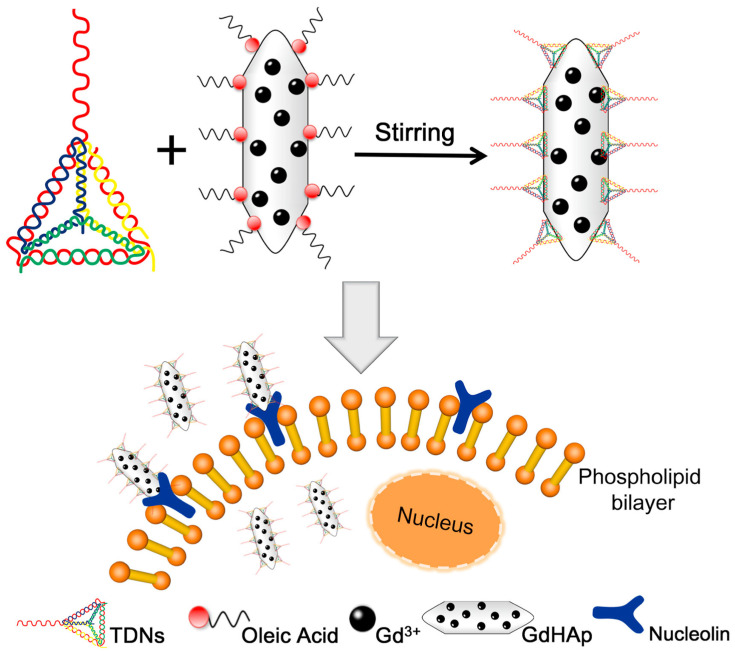
Synthesis and application process of the Apt-TDNs-Gd-HAp probe. Reprinted from [151] with the permission of Elsevier. Copyright © 2023.

More innovative designs involve the use of tetrahedral DNA nanostructures (TDNs) to construct monodisperse lanthanide-substituted HAp NPs. He et al. conjugated the TDNs with an anti-nucleolin overexpressed on tumor cell membranes (AS1411 aptamer) and the resultant nanocomposite exhibited a higher crystallinity, a longitudinal relaxivity two times higher than conventional Gd-HAp NPs and a more selective endocytosis to tumoral cells (Figure 12) [151]. These results also corroborated the benefit of using aptamers for the generation of site-selective and controlled delivery of drugs observed on MCF-7 cells treated with aptamer-capped Gd/Sr-HAp NPs [152].

Not all attempts to develop these systems have been successful. Methotrexate (MTX), an antitumoral drug, was loaded into a mesoporous Eu-HAp/poly(methacrylic acid) composite and drug release kinetic studies at pH 5 and 7 were performed. At physiological pH, a rapid release of MTX during the first 2 h was evidenced, reaching 63% of the loaded drug. A constant release followed this stage after 6 h. At pH 5, a trapping of MTX was registered, with only 0.5% of the pharmaceutical agent released during the first 2 h and 4% at around 16 h. This result demonstrated that even though the nanocomposite showed good drug loading efficiency, the delivery system was not adequate for cancer treatment due to the electrostatic interactions between the antitumoral agent and the components of the composite [153].

### 5.3. Lanthanides-Substituted HAp for the Diagnosis, Treatment or Control of Other Pathologies

Great developments in medical diagnosis have been achieved in recent years. As part of this progress, research has contributed to the elaboration of nano-sensors for the identification and quantification of different biomarkers. For example, Eu-HAp NPs were studied as possible nano-sensors of cysteine and homocysteine that were detected by fluorometric detection with a limit of 110 nM and 160 nM, respectively [154]. This method has also enabled the determination of the activity and inhibition of protein kinase A indirectly by detecting the presence of the phosphorylated peptide that quenches the fluorescence of the NPs [155].

Quantification of creatine was accomplished by luminescence spectroscopy with a nano-sensor based on Eu-ACP. The sensor proportionated a constant signal up to 4 months with a detection range of 1–120 µM and an optimal biomaterial concentration of 250 mg/L. The efficiency of the detector was demonstrated by running cycles of detection of the biomarker in citrate buffer or in human urine from healthy volunteers. In all cases, creatine and other molecules in the urine (e.g., glutamic acid, ascorbic acid, glucose or urea) did not interfere or affect the emission wavelengths, as the emission peak at 616 nm served as a quantification reference. The stability of the doping was assessed by the quantification of Eu^3+^ release and it was found that up to 3.6 wt.% was liberated after 4 months of storage [117].

Constituents of bacterial spores are included in the list of molecules detected by Eu-HAp-based nano-sensors. Li et al. detected dipicolinic acid from the bacterial spores of *B. subtilis,* with a detection limit of 77 nM [154].

Ce-HAp can be used to produce electrodes for the simultaneous detection of norepinephrine (NE), tyrosine (Tyr) and uric acid (UA). Kanchana et al. found that a glassy carbon electrode could be covered with a 5 at. % of Ce-doped HAp. The system showed a fast interfacial electron transfer and improved electrocatalytic behavior over pristine HAp and 3 at. % Ce-HAp. The system even showed a good capability to resolve the overlapped CV signals at 0.26, 0.42 and 0.77 V ascribed to NE, Tyr and UA. The detection limits of the electrode for each biomarker were 0.058, 0.072, and 0.39 µM, respectively. These results show a promising new field for doped HAp, but further studies are required to determine the detection precision in a more complex biological sample, such as urine or blood [26].

Lanthanide-doped HAp NPs can be used to treat a variety of pathologies. Current research has paid particular attention to the treatment of chronic synovitis associated with bleeding disorders. In detail, hemarthrosis is one of the most frequent clinical presentations of hemophilia. Patients diagnosed with this pathology can suffer from recurrent hemarthroses and chronic synovitis. If that is the case, radioactive synovectomy can be applied and consists of synovial ablation by injecting a beta-emitting radionuclide, such as yttrium (^90^Y), rhenium (^186^Re), dysprosium (^165^Dy), samarium (^153^Sm) and phosphorus (^32^P). One of the main problems that arises is the extra-articular leakage of small isotopes [156]. In order to overcome this situation, some authors have proposed linking the isotopes to HAp NPs, obtaining a reduction of the joint bleeds to similar levels as the isotopes alone, regardless of the treated joint, age, gender or radiologic state [156].

Radioactive synovectomy can be similarly used as an alternative treatment for rheumatic arthritis, commonly treated with an intra-articular injection of corticosteroids. In the case of ^153^Sm-HAp, the penetration of the β energy makes it appropriate for the synovectomy of median articulations. There have been attempts to use NPs for ankles, elbows and knees, obtaining opposite results. By increasing the dose to 185 or 740 MBq in clinical studies, an improvement of knee motility was reported with 740 MBq and a few cases of mild reactional synovitis were observed after one year of follow-up [157]. Manzzini et al. obtained better results when evaluating the response to synovectomy with ^153^Sm-HAp of the ankles or elbows of 82 patients with hemophilia [158]. A similar case was reported in a clinical study that included patients with persistent rheumatoid knee synovitis [159]. On the contrary, in a study with 62 patients and with a higher variety of diagnoses, such as rheumatoid arthritis, psoriatic arthritis, ankylosing spondylitis or reactive arthritis, no clear beneficial clinical effect was determined after one year of treatment [160]. The injection of ^153^Sm-HAp (15 mCi) with 40 mg of triamcinolone hexacetonide (TH) did not represent a superior treatment for knee synovitis in rheumatoid arthritis patients [52]. Altogether, it can be concluded that further studies are required to actually determine if the isotope association with HAp represents a real benefit for the patient and a better design for the clinical studies should be implemented. ^177^Lu-HAp and ^169^Er-HAp NPs have also been produced for this purpose [161,162].

Lanthanides-doped HAp can also be implemented as a drug carrier. La-HAp can be used to load amoxicillin, extending the release up to 34–76 h, depending on the substitution percentage by the La^3+^ (2–8.6%-wt.). In vitro, there was an initial burst release of 27–35% during the first 5–7 h, followed by a slower release rate. The encapsulation of the drug inside the doped HAp NPs increased the antibacterial resistance up to 64%, 20%, 40% and 42% more over the effect of pure HAp against *E. coli*, *S. aureus*, *Bacillus* and *Pseudomonas*, respectively [29].

The OH groups on the surface of HAp are proposed to be the reaction sites for forming hydrogen bonds with the carboxyl groups of different pharmaceutical agents. This is the case for ibuprofen that was loaded into Eu-HAp NPs with an efficiency of 46 wt.%. The release profile revealed a burst effect of about 50% in 1 h of monitoring and a complete release at 24 h [55].

### 5.4. Lanthanides-Substituted HAp for Biolabeling and Bioimaging

Jadalannagari et al. developed La-HAp NPs (*x* = 0.02, 0.06 and 0.1) that were internalized by human embryonic kidney and human adenocarcinoma cells. Under tetramethylrhodamine (TRITC) and fluorescein (FITC) filters in epifluorescence microscopy, the cells demonstrated fluorescence after NP internalization. Lower cell viability and internalization values were obtained with increasing La concentrations [28].

The potential of Eu-HAp NPs for cell labeling was demonstrated in Bel-7402 human liver cancer [163]. Liu et al. synthetized Eu-HAp NPs and managed to tract the relative position of an Eu-HAp scaffold and the treated cells based on luminescence imaging from the doping [56]. In the same line of research, Krishnapriya et al. made eggshell-derived HAp NPs doped with Eu^3+^ at 2,5,10,15 and 20 at. %. They determined that the best doping concentration for in vivo imaging was 15% [67].

Paramagnetic properties can be proportionated to Eu-HAp NPs by co-doping with Dy^3+^. As mentioned in other Sections, Eu3^+^ photoluminescence demonstrated a concentration dependent behavior and Dy^3+^ addition enhanced the luminescence properties of the ion due to an energy transfer from Dy^3+^ to Eu^3+^. The NPs did not show a toxic profile on L929. Based on good results, this material was proposed as a contrast agent for MRI in functional implant coatings that would allow the live tracking of tissue repair [164].

To summarize this Section, the biomedical applications of lanthanides-substituted HAp are presented by categories in Table 2. In the case of praseodymium, despite the number of studies that evaluated its potential biological role and antimicrobial effect, a clear biomedical application of HAp doped with this ion was not found during the elaboration of this review.

## 6. Substituting HAp with Lanthanides: Possible Economic Impact in the Synthesis Process and the Cost of the Material

Lanthanides are rare earth elements with atomic numbers from 57 to 71 and, generally, those with low atomic numbers are more abundant in the earth crust. Rare earth mines operated in South Africa, India and Brazil in the 1950s. From the 1960s to 1980s, the largest global producer was a mine in Mountain Pass, California. In the 1990s, China began a large-scale production and exportation of cheaper rare earth elements. As the Mountain Pass mine shut down in 2002, China is currently the world’s largest producer of rare earth elements, providing more than 95% of the world’s total supply from its mines in Inner Mongolia [165,166].

Lanthanides are becoming indispensable in high-tech industries, such as hybrid cars, wind turbines, mobile phones and fluorescent light. Certain applications even required a combination of them, such as the use of Nd and Pr for rare earth magnets and Eu and Y for rare earth phosphors. Nevertheless, despite the increased demand for these elements, the preparation of high-purity lanthanides must be taken into consideration to evaluate how incorporating these elements into HAp can impact the final cost of the materials for the biomedical industry. In general, lanthanides can be obtained by solvent extraction methods, namely cation exchangers, solvation extractants and anion exchangers. All these processes imply that up to hundreds of stages of mixers and settlers must be assembled to separate the lanthanides individually, which can increase the cost of extraction and selling [165]. Together with a limited offer due to a reduced number of operative mines, they could impact the price in the international market. 

It can be concluded that, although the future of lanthanides in the science and biomedical fields look promising based on the biological effects of the elements and the properties that they can endow to different biomaterials, special consideration should be given to the possible effect of their use in the biomedical industry and the cost of the medical scaffolds and carriers.

## 7. Conclusions

HAp is a ceramic of preference in biomedical research, as it represents the main inorganic component of the hard tissues, it can promote cell proliferation, and it can encapsulate a variety of pharmaceutical agents and biomolecules for targeted therapy. Nevertheless, it has low mechanical properties and a lack of antimicrobial effects, which can limit its application. In an attempt to improve its properties, different cationic ions have been used as dopant agents. Interestingly, recent research has mainly focused on the evaluation of the biomedical applications of specific cationic ions such as Sr^2+^, Ag^+^ or Zn^2+^, forgetting the potential biological role of other cationic ions, such as lanthanides. The present article reviews the effect of these elements on HAp structure and properties, but, more importantly, it summarizes the biological role of the ions in order to bring a light on the applications that have not yet been exploited. Substitutions by lanthanides can endow the calcium phosphate with an increased hardness, photoluminescence properties, increased electrical conductivity and a lower dissolution in PBS. Nevertheless, their incorporation can hinder crystallinity and induce the formation of secondary phases, such as β-TCP. From a biological perspective, doping HAp with lanthanides can improve osteoconductivity and angiogenesis, decrease osteoclastogenesis, reduce ROS formation, promote the anti-inflammatory macrophage phenotype (M2) and endow HAp with antimicrobial properties. Based on these characteristics, it can be concluded that there are several potential applications of lanthanides-substituted HAp in the biomedical field, such as drug carriers, bone fillers or platforms for theragnostics. Finally, it is important to emphasize the need to further evaluate the permitted concentration range for doping HAp with these elements without reaching cytotoxic levels. In addition, there is a strong necessity to not only analyze the effects of NPs and scaffolds using an in vitro approach.

## Figures and Tables

**Table 2:** Biomedical applications of lanthanides-substituted HAp.

Lanthanide	Biomedical Application	Reference
Lanthanum	Implant coating for antibacterial properties and improved osteogenesis Regulator of the macrophage’s phenotype toward the anti-inflammatory M2 Drug carrier Biolabeling/bioimaging	[28,29,36,38,88,138]
Cerium	Implant coating for antibacterial properties, improved mechanical properties, anticorrosion effect and osteoconductive Bone regeneration material	[26,44,45,48,103,106,139,141]
Samarium	Implant coating for antibacterial properties, anticorrosion effect and osteoconductive Theragnostics Biomarker’s detection and quantification Radioactive synovectomy	[52,143,156,157,158,159,160]
Europium	Implant coating for antibacterial properties, anticorrosion effect and osteoconductive Theragnostics Drug carrier Biolabeling/bioimaging Biomarker’s detection and quantification Bacterial detection	[55,56,58,67,117,144,153,154,155,163,164]
Neodymium	Theragnostics	[71,72]
Gadolinium	Implant coating for antibacterial properties, anticorrosion effect and osteoconductive Artificial bone filler Theragnostics	[73,126,127,143,148,150,151,152]
Dysprosium	Theragnostics Radioactive synovectomy Photoluminescence enhancer for biolabeling/bio-imaging	[147,156,164]
Erbium	Radioactive synovectomy	[162]
Lutetium	Radioactive synovectomy	[161]
Ytterbium	Bone regeneration based on osteoconductive and angiogenic potential Radioactive synovectomy	[145]

## Data Availability

Not applicable.
